# Right Ventricular Outflow Tract Reconstruction in Truncus Arteriosus: A 30-Year Two-Center Comparison between Homografts and Bovine Jugular Vein

**DOI:** 10.21470/1678-9741-2022-0341

**Published:** 2023-07-18

**Authors:** Ventsislav Boshnakov, Ivaylo Mitev, Stojan Lazarov, Dimitar Pechilkov, Beatrice Desnous, Fedoua El Louali, Loic Macé, Virginie Fouilloux, Marien Lenoir

**Affiliations:** 1 Department of Congenital Heart Surgery, National Heart Hospital, Sofia, Bulgaria; 2 Pediatric Cardiac Intensive Care Unit, National Heart Hospital, Sofia, Bulgaria; 3 Department of Pediatric Neurology, La Timone Children Hospital, Marseille, France; 4 Department of Pediatric Cardiology, La Timone Children Hospital, Marseille, France; 5 Department of Congenital Heart Surgery, La Timone Children Hospital, Marseille, France

**Keywords:** Jugular Veins, Persistent Truncus Arteriosus, Allografts, Reoperation, Catheters

## Abstract

**Introduction:**

Homografts and bovine jugular vein are the most commonly used conduits for right ventricular outflow tract reconstruction at the time of primary repair of truncus arteriosus.

**Methods:**

We reviewed all truncus patients from 1990 to 2020 in two mid-volume centers. Inclusion criteria were primary repair, age under one year, and implantation of either homograft or bovine jugular vein. Kaplan-Meier analysis was used to estimate survival, freedom from reoperation on right ventricular outflow tract, and freedom from right ventricular outflow tract reoperation or catheter intervention.

**Results:**

Seventy-three patients met the inclusion criteria, homografts were implanted in 31, and bovine jugular vein in 42. There was no difference in preoperative characteristics between the two groups. There were 25/73 (34%) early postoperative deaths and no late deaths. Follow-up for survivals was 17.5 (interquartile range 13.5) years for homograft group, and 11.5 (interquartile range 8.5) years for bovine jugular vein group (*P*=0.002). Freedom from reoperation on right ventricular outflow tract at one, five, and 10 years in the homograft group were 100%, 83%, and 53%; and in bovine jugular vein group, it was 100%, 85%, and 50% (*P*=0.79). There was no difference in freedom from reoperation or catheter intervention (*P*=0.32).

**Conclusion:**

Bovine jugular vein was equivalent to homografts up to 10 years in terms of survival and freedom from right ventricular outflow tract reoperation or catheter intervention. The choice of either valved conduit did not influence the durability of the right ventricle-pulmonary artery conduit in truncus arteriosus.

## INTRODUCTION

Truncus arteriosus (TA) was first reported by Wilson in 1798 in an autopsy case. In 1864, Bauchanan^[1]^ described its anatomical details in a six-month-old infant. TA is a rare disease with incidence of < 0.35% of all congenital heart diseases^[2]^, but it accounts for 4% of all critical congenital heart diseases. Because of its specific anatomical and hemodynamic features, early development of severe pulmonary hypertension and truncal valve (TV) dysfunction, natural evolution results in high mortality — up to 80%^[3]^ until the age of one year. The contemporary treatment of TA is a single-staged surgical repair in the neonatal period, or in the first few months. One of the most challenging parts remains the right ventricular outflow tract (RVOT) reconstruction. Since the first successful surgical repair using aortic homograft (HG) in 1968 by McGoon^[4]^, a lot of improvements in surgical technique and perioperative management has occurred. Nevertheless, the overall mortality remains high — 3-20% with long-term survival of approximately 75% at 20 years^[3]^. Concerning the RVOT reconstruction, various surgical methods without an accepted standard are currently used — direct right ventricle to pulmonary artery (RV-PA) anastomosis, HG, or other type of valved conduit implantation^[5,6,7,8]^. In 2021, a consensus document on optimal management of patients with TA was issued^[8]^. Regardless the type of conduit at the time of initial repair, a reoperation is required for its replacement when child grows up. The reasons are small sizes used in the first weeks of life, limited durability, and degeneration. The most widely used valved conduits nowadays are HGs, followed by bovine jugular vein (BJV) (Contegra™, Medtronic, Minneapolis, Minnesota, United States of America). The BJV is reported to have good handling characteristics but inferior freedom from degeneration and reintervention^[6]^. This study aims to analyze patients after primary repair of TA with HG or BJV implantation, in terms of survival, freedom from RVOT reoperations, and freedom from RVOT reoperation or catheter intervention.

## METHODS

### Study Population

The medical records of all patients in the congenital heart surgery of National Heart Hospital (Sofia, Bulgaria) and La Timone Children Hospital (Marseille, France) who underwent primary truncus repair under one year of age, from January 1990 to December 2020, were retrospectively reviewed (n=80). Additional inclusion criterium was the implantation of either HG or BJV. Seven patients were excluded (n=7; two operated with direct RV-PA anastomosis, one received non-valved Gore-Tex® [W.L. Gore & Assoc, Flagstaff, Arizona, United States of America] tube, and four received Hancock® conduits [Medtronic, Minneapolis, Minnesota, United States of America]). Follow-up echocardiography and exam reports were obtained from hospital record systems.

The study was approved by institutional review boards in both hospitals (TPL7AP) and the need for patient consent was waived due to the study’s retrospective nature. Derived data supporting the findings are available from the corresponding author on request.

### Patients and Definitions

Early in-hospital mortality was defined as occurring within 30 days of surgery or before hospital discharge. All other deaths were considered late. Intensive care unit (ICU) length of stay was calculated as the number of calendar days from the day of admission (counted as one day) to the day of ICU discharge. The time from the surgery date to the date of death, or last follow-up, was considered the patient survival time. This study focused on longevity of RV-PA conduits, therefore only conduit associated reinterventions were included. Reoperations included all RVOT surgical procedures, regardless TV surgery. Catheter interventions included all catheter-based procedures related to RVOT. The time from the surgery date to the date of reoperation/catheter intervention was considered the time of freedom from RVOT reoperation or catheter intervention.

### Surgical Technique

All patients underwent median sternotomy, standard cardiopulmonary bypass (CPB) with mild hypothermia, or deep hypothermic circulatory arrest. Intracardiac repair was performed during aortic cross-clamping with cold blood cardioplegia (CP1B AP-HP solution, that consists of magnesium, potassium, and procaine, added to oxygenated blood in 3:1 ratio) (Marseille) or crystalloid cardioplegia (Sofia). All BJV conduits were washed in normal saline at least three times before implantation. The pulmonary artery was separated from the truncal root and extensively mobilized. The distal end was cut as short as possible in order to proper positioning the conduit, while a right ventriculotomy was then performed as high as possible within a safe distance of the main coronary arteries. The ventricular septal defect closure was performed using a heterologous pericardial patch or Dacron® patch through the right ventriculotomy or through the TV. In HG implantation, the proximal anastomosis was augmented with a hood of autologous pericardium, Gore-Tex®, or anterior mitral leaflet in aortic HGs. All HGs were cryopreserved with the same conservation technique during the study period^[7]^. In almost all of the cases, smallest conduits available were used.

### Patient Assessment and Follow-up

All patients were assessed preoperatively, postoperatively, at discharge and annually after surgery. Routine baseline diagnostic examination included:

1. Clinical examination – oxygen saturation, blood pressure, heart murmur, and signs of heart failure
2. Electrocardiogram – standard 12-lead electrocardiogram
3. Transthoracic echocardiography with focus on the RV-PA conduit dimensions and function, assessed in the parasternal long-axis, parasternal short-axis, and subcostal view. Conduit stenosis was assessed by measurement of peak velocity across the pulmonary valve using continuous-wave Doppler and applying the modified Bernoulli equation to calculate the peak gradient. Trace of the full envelope was used to calculate the mean pressure gradient. Color Doppler and pulsed-wave Doppler were used to evaluate the pulmonary regurgitation. In all of the cases in which reintervention was considered, a cardiovascular magnetic resonance imaging and/or cardiac catheterization was done.

As stated in the 2021 consensus, the timing for conduit replacement in TA is no different than in other situations, and is based on a composite indication of raised right ventricular (RV) pressures (> 67% systemic pressure in the right ventricle), impaired RV function, and the development of exercise limitation associated with conduit stenosis or impaired function^[8]^.

### Outcomes

Primary endpoints of interest were defined: survival, freedom from RVOT reoperation, and freedom from RVOT reoperation or catheter intervention in both groups.

Secondary endpoints were focused on patients’ perioperative variables — CPB time, aortic cross-clamping time, delayed sternal closure (DSC), ICU length of stay, mechanical ventilation time, conduit size, and conduit size/body surface area (BSA) index.

### Statistical Methods

Data are expressed as mean ± standard deviation for normally distributed continuous variables, or median and interquartile range (IQR) for non-normally distributed continuous variables; frequency and percentage (%) were used for categorical variables. The Kolmogorov-Smirnov test was used to determine normal distribution. For continuous variables, comparisons between the groups were made using Student’s t-tests or Mann-Whitney U test, as appropriate. Fisher’s exact tests were used for comparisons of categorical variables. Survival, freedom from RVOT reoperation, and freedom from RVOT reoperation or catheter intervention were displayed graphically using Kaplan–Meier curves, and log-rank test was used for comparison between groups. An adjustment for multiple tests was not used. *P*-values < 0.05 were considered statistically significant. All data were analyzed using statistical software IBM Corp. Released 2011, IBM SPSS Statistics for Windows, version 20.0, Armonk, NY: IBM Corp.

## RESULTS

A total of 73 patients met the inclusion criteria; 31 patients had HG implanted, compared to 42 patients with BJV. HGs were the preferable option in Marseille (n=30/31 HG patients). BJV was most commonly used in Sofia (n=30/42 BJV patients), due to lack of availability of small HG in Bulgaria.

Patient preoperative characteristics are summarized in Table 1. Median age at surgery was 50 days (IQR 75) in the HG group, and 77.5 days (IQR 89.5) in the BJV group. There were no significant differences in age at surgery (*P*=0.15), weight (*P*=0.07), sex (*P*=0.18), type of TA (*P*=0.15), presence of coronary anomalies (*P*=0.26), DiGeorge syndrome (*P*=0.23), severity of TV regurgitation (*P*=0.96), or in the anatomy of TV cusps (*P*=0.58).

**Table 1:** Patient preoperative characteristics.

Variables	HG group (N=31 pts)	BJV group (N=42 pts)	*P*-value
Age at surgery (days), median (IQR)	50 (75)	77.5 (89.5)	0.15
Weight at surgery (kg), median (IQR)	3.45 (1.1)	3.65 (1.88)	0.22
Female, n (%)	14 (45%)	16 (38%)	0.18
DiGeorge syndrome, n (%)	5 (16.1%)	4 (9.5%)	0.23
Van Praagh classification, n (%)			0.15
Type I	12 (38.7%)	23 (54.8%)	
Type II	16 (51.6%)	13 (31%)	
Type III	0 (0%)	3 (7.1%)	
Type IV	3 (9.7%)	3 (7.1%)	
Coronary anomalies, n (%)	3 (9.7%)	8 (19%)	0.26
TV regurgitation ≥ 3, n (%)	9 (29%)	12 (28.6%)	0.96
TV cusps anatomy, n (%)			0.58
Bicuspid	1 (3.2%)	5 (12%)	
Tricuspid	19 (61.3%)	23 (54.8%)	
Quadricuspid	11 (35.5%)	14 (33.3%)	

BJV=bovine jugular vein; HG=homograft; IQR=interquartile range; TV=truncal valve

Perioperative variables are summarized in Table 2. The median conduit size at the time of implantation was 13 mm (IQR 2) for HG *vs.* 12 mm (IQR 2) for BJV (*P*=0,025). When comparing conduit size/BSA, HG group showed a mean of 60 mm/m2 ±14 in the BJV group, it was 54 mm/m2 ±16 (*P*=0.004). There were no significant differences in CPB time (*P*=0.51), aortic cross-clamping time (*P*=0.99), DSC (*P*=0.98), ICU length of stay(*P*=074), hospital length of stay (*P*=0.44), and mechanical ventilation time (*P*=0.39).

**Table 2:** Perioperative variables.

Variables	Homograft (N=31 pts)	BJV group (N=42 pts)	*P*-value
In-hospital death, n (%)	12 (38%)	13 (30%)	0.49
CPB (min), median (IQR)	168 (59)	176 (61.25)	0.51
Aortic cross-clamping time (min), median (IQR)	93 (24)	92 (37.5)	0.991
Conduit size (mm), median (IQR)	13 (2)	12 (2)	0.025
Conduit size/BSA, median (IQR)	60 (14)	54 (16)	0.004
DSC (days), median (IQR)	3 (4)	2 (3)	0.98
ICU length of stay (days), median (IQR)	10 (9)	8.5 (9.5)	0.74
Hospital length of stay (days), median (IQR)	19 (25)	21 (19.5)	0.44
Mechanical ventilation (hours), median (IQR)	192 (168)	144 (145)	0.39

BJV=bovine jugular vein; BSA=body surface area; CPB=cardiopulmonary bypass; DSC=delayed sternal closure; ICU=intensive care unit; IQR=interquartile range

Overall in-hospital mortality was 34.2% (n=25/73) — 39% in HG group (n=12), 31% in BJV group (n=13) — with no difference between the groups (*P*=0.45). Mortality was comprised only of early postoperative mortality at primary repair. Causes of in-hospital mortality include low cardiac output syndrome (n=9), pulmonary hypertension (n=5), coronary ischemia (n=2), hemorrhagic shock (n=1), and unknown (n=8). There were no late deaths.

**Fig. 1:**
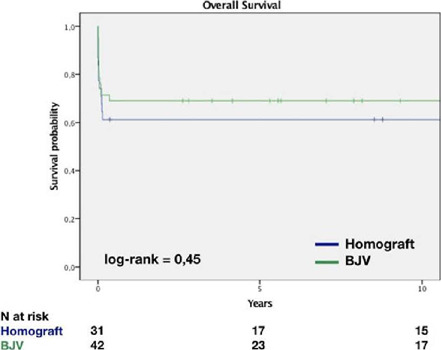
Overall survival. BJV=bovine jugular vein.

Kaplan-Meier estimates of overall survival at one, five, and 10 years were constant with no significant difference between the groups: 61±8% in HG group and 69±7% in BJV group (*P*=0.45) (Figure 1).

A total of 19 survivals (19/48; 40%) required conduit replacement (HG n=8, BJV n=11). There were no deaths at time of replacement, and there were no reinterventions due to infective endocarditis.

**Fig. 2:**
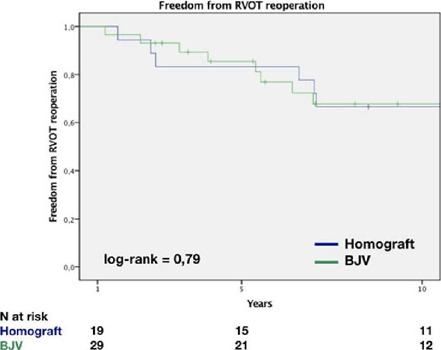
Freedom from right ventricular outflow tract (RVOT) reoperation. BJV=bovine jugular vein.

At the time of first redo, among the HG group, seven patients received HG again, and one Contegra™. In the BJV group, seven patients received Contegra™, one HG, and three porcine-valved Dacron® conduits. Median time of a redo was 6.4 (IQR 7.93) years for all survivals. Freedom from reoperation was 83±8% and 85±6% at five years and 53±12% and 50±11% at 10 years for HG and BJV, respectively (*P*=0.79) (Figure 2).

**Fig. 3:**
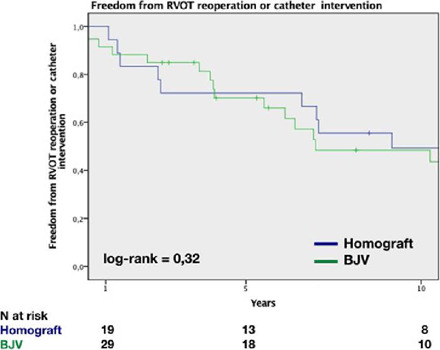
Freedom from right ventricular outflow tract (RVOT ) reoperation or catheter intervention. BJV=bovine jugular vein.

Freedom from RVOT reoperation or catheter intervention has been used to assess overall durability of conduits. Catheter interventions were required in 14 patients. A total of seven patients required balloon dilatation, and a total of seven stents and two Melody® valves (Medtronic, Minneapolis, Minnesota, United States of America) were inserted. Freedom from RVOT reoperation or catheter intervention was 94±5% at one year, 72±10% at five years, and 49±11% at 10 years in the HG group, and 91±4%, 70±8%, and 51±10% at one, five, and at 10 years, respectively, in the BJV group (*P*=0.32) (Figure 3).

### Follow-up

Follow-up for survivals was 17.5 (IQR 13.5) years for the HG group and 11.5 (IQR 8.5) years for the BJV group (*P*=0.002). At the last follow-up, all patients were in New York Heart Association (or NYHA) functional class I or II.

## DISCUSSION

This is a 30-year retrospective, observational, two-European center study, reviewing RVOT reconstruction in TA. Despite the differences in healthcare systems, both centers in Marseille and Sofia are mid-volume surgical programs (~150-300 CPB per year). This favored a study comparing RVOT conduits used at the time of primary repair. Contegra™ became available in Europe in 1999, which means there were no BJV patients in the first decade of our study. This time difference resulted in significant follow-up difference and limited our study to a 10-year comparison.

Implantation of any type of RVOT conduit under one year of age will always question its durability, since in a growing child several reoperations are inevitable to adjust the graft size. Recently published consensus on truncus patients management by task forces of the European Association for Cardio-Thoracic Surgery and Association for European Paediatric and Congenital Cardiology encompassed the full spectrum of the disease, including the reconstruction of RVOT^[8]^. No evidence supports valved strategy over direct anastomosis in terms of survival or operative outcome^[9]^. Although recently Derridj et al.^[10]^ showed strong evidence with excellent results with direct RV-PA anastomosis using left atrial appendage, valved conduits are still the most prevalent technique used in the literature^[11]^. Naimo et al.^[12]^ published a 40-year experience with 239 patients who underwent conduit repair and 16 direct anastomosis. The rate of RVOT reoperations was high with freedom from reoperation at 10 years, 28.5% for conduit and 35.5% for direct anastomosis. After its first description by McGoon in 1968, HG were thought to be the ideal conduit for RVOT reconstruction. In the recent consensus, pulmonary and aortic HG have been qualified as best and second-best performance, while BJVs perform “generally good but inferior to homografts”^[8]^. First ever retrospective comparison of BJV conduit and down-sized pulmonary HG by Bove^[13]^ showed comparable results between two conduits. In 2008, Protopapas reviewed 17 articles with 767 patients related to RVOT reconstruction with BJV and found contradictory results. In some series, patients with BJV had higher incidence of stenosis and short-term reintervention when small sizes were used (12-14 mm), while other authors were very BJV-enthusiasthic^[14]^. Myens reported unsatisfactory results with excessive proliferation of neointima at the level of the distal anastomosis and stopped utilizing Contegra™^[15]^, while Fiore recommend BJV conduit as a first choice for conduit replacement in patients < 2 years of age^[16]^. In a similar European/American two-center study, BJV showed inferior to aortic/pulmonary HGs in time to conduit failure^[17]^. Center, conduit modification, and bypass or cross-clamping time at implantation did not influence conduit longevity. Our study confirms these results with no difference in perioperative data. These contradictory results may be partially explained by the heterogeneity of the series, surgery techniques, and variety of indications.

Both conduits have their advantages and disadvantages. Small HGs have very limited availability, and in Bulgaria, HG lack in general. Preservation and storage technique of HGs could influence the strength of immune response, as well as their quality. HG degeneration and calcification result from reaction of tissue rejection^[18]^.

In BJV, the length of a jugular vein valve is much longer, which is an issue in neonates where the distance between RV and PA is very short. The principal site of BJV conduit obstruction has been proven to be at the distal anastomosis with neointimal proliferation. The origin is suggested to be inadequate glutaraldehyde removal, immune response^[18]^, or chronic trauma to neointima, resulting in different series from 6% to 50% of hemodynamically significant supravalvular stenosis^[15,19]^. Nevertheless, distal anastomotic stenosis was also reported with HG in the Ross series^[20]^ and when using Gore-Tex® non-valved conduits in TA repairs.

In our series, after successful postoperative hospital discharge, survival was excellent. The high early mortality was probably related to late indication for surgery — median age of primary repair was about two months. With the methods of prenatal and early diagnosis we have in most cases today, we aim to perform exclusively neonatal surgery. Also, RV-PA conduit diameter > 50 mm/m^2^ were identified as an independent risk factor increasing five-fold mortality by Mastropietro et al.^[21]^; both of our groups showed a median diameter > 50 mm/m^2^. Oversized conduits require larger ventriculotomy and could increase risk for coronary artery compression. Unfortunately, small-sized conduits aren’t always available.

We were not able to prove any influence of age, body weight, length of CPB, and type of conduit to mortality. Furthermore, during the study period, Sofia center had no opportunity to utilize extended mechanical circulatory support. Use of ECMO in patients with low cardiac output might improve the reported mortality.

Since the fate of TV will be a subject of a different review, in our study we included only reinterventions associated to RV-PA conduit. Timing for conduit replacement was no different from in other situations, and was based on indications of raised RV pressure, impaired RV function, or other conduit dysfunction^[8]^. While freedom from redo/catheter intervention did not differ significantly between the two groups, it revealed that about 30% of conduits will be changed by the 5^th^ year. Theoretically, timely catheter interventions could postpone surgery and increase lifespan of a conduit.

According to current guidelines, antibiotic infective endocarditis prophylaxis is recommended in all types of cyanotic congenital heart disease, moreover in patie nts repaired with a prosthetic material like RV-PA conduits^[22]^. Fortunately, we didn’t observe any reintervention due to infective endocarditis.

We have demonstrated comparable results between HG and BJV groups. As both conduits showed gradual deterioration with time, regular follow-up and timely referring to surgery or intervention is an important detail in management in these patients^[22,23,24,25]^. Particular emphasis in echocardiography examination should be conduit valve function and any gradients on proximal or distal anastomotic site.

### Limitations

Our small number of cases and small number of events might generate certain biases. Data were acquired retrospectively; however, this is an accepted study design when dealing with low-incidence disease. The operations were performed by different surgeons in Marseille and Sofia, who chose the conduit type by their preference or availability, not by randomization. Perioperative management strategies in both centers were standardized but could have significant differences with impact on patient outcome. Aortic and pulmonary HG data have been collected in the same group, however, they could perform with different results.

## CONCLUSION

Our two-center independent results add to the current knowledge surrounding the RVOT reconstruction in truncus patients, showing that BJV conduit was equivalent to HG up to 10 years for survival, freedom from RVOT reoperation, and freedom from RVOT reoperation or catheter intervention. Commercially available Contegra™ is a good alternative to HGs, when the latter are unavailable. The choice of either valved conduit did not influence the durability of the RV-PA conduit in truncus arteriosus. A randomized multi-center controlled trial with long-term follow-up is still needed to solve the HG/BJV debate.

## Abbreviations, Acronyms & Symbols

BJV: Bovine jugular vein
BSA: Body surface area
CPB: Cardiopulmonary bypass
DSC: Delayed sternal closure
HG: Homograft
ICU: Intensive care unit
IQR: Interquartile range
RV: Right ventricular
R V- PA: Right ventricle to pulmonary artery
RVOT: Right ventricular outflow tract
TA: Truncus arteriosus
TV: Truncal valve
